# Ethanol vapor-driven multicarbon chemistry enables high-performance Mg–CO_2_ battery

**DOI:** 10.1093/nsr/nwaf330

**Published:** 2025-08-12

**Authors:** Wenbo Liu, Lu Li, Menggang Li, Ning Wang, Yanmei Li, Zongqiang Sun, Youxing Liu, Mingyang Chen, Rui Xu, Shaojun Guo

**Affiliations:** School of Materials Science and Engineering, University of Science and Technology Beijing, Beijing 100083, China; School of Materials Science and Engineering, Peking University, Beijing 100871, China; School of Materials Science and Engineering, Peking University, Beijing 100871, China; School of Materials Science and Engineering, Peking University, Beijing 100871, China; School of Materials Science and Engineering, University of Science and Technology Beijing, Beijing 100083, China; School of Materials Science and Engineering, University of Science and Technology Beijing, Beijing 100083, China; School of Materials Science and Engineering, Peking University, Beijing 100871, China; School of Materials Science and Engineering, Peking University, Beijing 100871, China; School of Materials Science and Engineering, University of Science and Technology Beijing, Beijing 100083, China; School of Materials Science and Engineering, University of Science and Technology Beijing, Beijing 100083, China; School of Materials Science and Engineering, Peking University, Beijing 100871, China

**Keywords:** Mg–CO_2_ battery, ethanol vapor, multicarbon chemistry, long cycle life

## Abstract

Recent advancements in metal–CO_2_ batteries with enhanced energy efficiency in a sustainable manner largely rely on catalytic improvements to accelerate the sluggish kinetics of the CO_2_ reduction and evolution reactions (CO_2_RR/CO_2_ER) occurring at the multiphase interface. However, conventional solid and liquid catalysts often increase the adsorption of both reactant gas and solid-phase discharge products, lowering discharge overpotentials but impeding product decomposition during charging. This trade-off complicates efforts to simultaneously reduce charge–discharge overpotentials and improve overall battery efficiency. Here, we introduce an ethanol vapor-driven strategy for Mg–CO_2_ batteries—distinct from traditional solid and liquid catalysis—that selectively enhances CO_2_ adsorption while limiting the adsorption of discharge products. This approach enables high energy efficiency through the formation and decomposition of the first conductive organic multicarbon (C_2+_) product in metal–CO_2_ batteries, specifically Mg(CH_3_COO)_2_·4H_2_O. The Mg–CO_2_ battery delivers outstanding discharge and charge capacities beyond 50 000 mAh g^−1^, coupled with stable cycling over 600 h, ranking it the best Mg–CO_2_ system reported to date. This catalyst-free strategy for multicarbon production holds potential for applications in CO_2_ reduction and carbon fixation.

## Teaser

This work presents an ethanol vaporâ€“driven Mg-CO2 battery strategy that selectively enhances CO2 adsorption and suppresses discharge product adsorption, enabling the reversible formation of Mg(CH3COO)2â€¢4H2O, delivering over 600 h stable cycling.

## INTRODUCTION

Metal–CO_2_ batteries have attracted considerable attention for their ability to utilize the greenhouse gas CO_2_ to generate electricity in a sustainability manner, achieving an energy density comparable to that of petroleum [1–7]. Among various metal–CO_2_ batteries (e.g. Li, Na, K), rechargeable Mg–CO_2_ batteries have attracted increasing attention due to their high energy density, safety and sustainability [8–12]. Magnesium combines a high volumetric capacity (3833 mAh cm**^−^**^3^) with natural abundance (∼2.9% of Earth's crust), offering both compact CO_2_ storage potential and cost advantages [13,14]. Moreover, the intrinsic dendrite-free nature of the Mg anode enhances safety and ensures stable operation over prolonged cycling, making Mg–CO_2_ systems a compelling platform for next-generation carbon-utilizing energy storage [15–17]. Additionally, Mg–CO_2_ batteries hold great promise for future applications in marine energy systems and industrial environments, such as onboard power supplies for ships or as integrated systems for the capture and utilization of CO_2_ emissions directly from steel blast furnace chimneys.

In a typical reversible reaction within Mg–CO_2_ batteries, CO_2_ is reduced during discharge to form carbonates and carbon, while charging regenerates CO_2_, thereby storing energy [8,10,18,19]. Currently, advancements in Mg–CO_2_ batteries with higher energy efficiency largely hinge on catalytic improvements to accelerate the sluggish kinetics of CO_2_RR/CO_2_ER at the cathode. Although solid and liquid catalysts can reduce discharge overpotentials via an increase in the adsorption of CO_2_ and solid-phase discharge products, it complicates product decomposition during the charge process. This trade-off poses a grand challenge in simultaneously lowering charge and discharge overpotentials, which is crucial for improving battery energy efficiency.

Solid catalysts exhibit high structural integrity and well-defined active sites, enabling stable catalytic performance in Mg–CO_2_ batteries. Nevertheless, they still face challenges such as sluggish charge and mass transport at the solid–liquid–gas multiphase interfaces, which exacerbate voltage hysteresis. Additionally, their active sites can be deactivated by dense, insoluble and insulating carbonate products that accumulate on their surfaces. Liquid catalysts, on the other hand, provide better interfacial contact with the electrolyte and CO_2_ throughout the charge and discharge processes. However, their catalytic activity tends to degrade over extended operation and the cathode is still prone to increasing the impedance due to passivation from insulating deposits, resulting in diminishing efficiency over time. To address these challenges, it is essential to develop novel chemistries in a Mg–CO_2_ battery that (i) enhance CO_2_ adsorption while minimizing the adsorption of CO_2_RR products at the cathode surface and (ii) generate discharge products with inherent conductivity. The first strategy aims to reduce overpotentials in both the charge and the discharge processes—a balance that remains challenging to achieve in the field—while the second strategy seeks to maintain rapid charge transfer at the multiphase interface throughout the charge and discharge processes.

**Figure 1.**
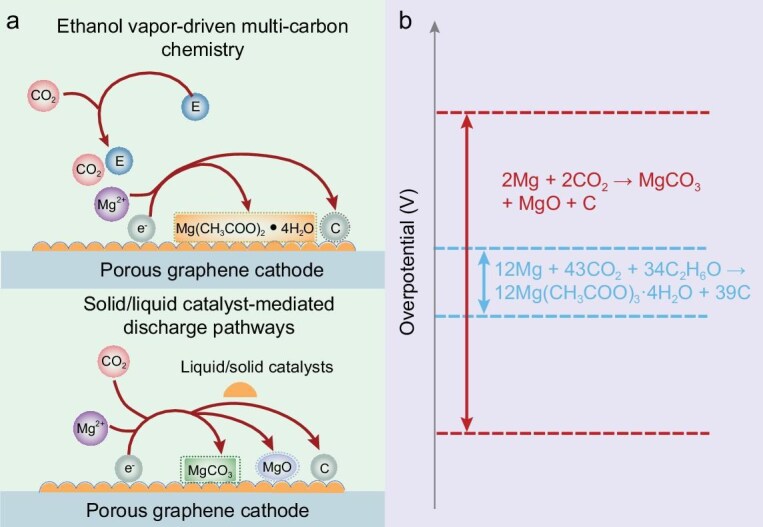
Schematic discharge reactions in Mg–CO_2_ batteries with or without ethanol vapor. (a) Electrochemical reaction paths and (b) the corresponding overpotential.

Herein, we present catalyst-free chemistry for Mg–CO_2_ batteries that meets these goals, demonstrates high round-trip efficiency and extends the cycle life. By introducing ethanol vapor into the CO_2_ atmosphere, we circumvent the conventional MgCO_3_ and MgO reaction pathways, achieving the highly reversible formation and decomposition of a C_2+_ discharge product, Mg(CH_3_COO)_2_·4H_2_O (Fig. 1). Theoretical calculations suggest that ethanol vapor, via a self-oxidation reaction, enhances CO_2_ adsorption onto the graphene cathode while reducing the adsorption of Mg(CH_3_COO)_2_·4H_2_O, significantly lowering the energy barriers for both CO_2_RR and CO_2_ER in the discharge and charge processes. Additionally, the conductive nature of the Mg(CH_3_COO)_2_·4H_2_O discharge product, partly due to its thin-film morphology, facilitates charge transfer and prevents cathode passivation. The Mg–CO_2_ battery with ethanol (200 ppm) delivers capacities exceeding 50 000 mAh g^−1^ in both discharge and charge, and sustains over 600 h of stable cycling, representing the best performance among all reported Mg-CO_2_ batteries (Table S1).

## RESULTS AND DISCUSSION

### Electrochemical performance of Mg–CO_2_ batteries

**Figure 2.**
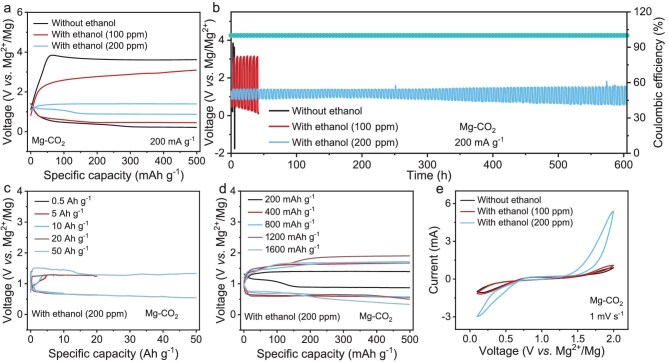
Performances of Mg–CO_2_ batteries. (a) Galvanostatic discharge–charge profiles of Mg–CO_2_ batteries by adding different contents of ethanol vapor. (b) Voltage–time curves of Mg–CO_2_ batteries by adding different contents of ethanol vapor. (c) Deep discharge–charge profiles of Mg–CO_2_ batteries with 200 ppm of ethanol vapor. (d) Discharge–charge profiles of Mg–CO_2_ batteries with 200 ppm of ethanol vapor under current densities from 200 to 1600 mA g^−1^. (e) CV curves of Mg–CO_2_ batteries by adding different contents of ethanol under a scan rate of 1 mV s^−1^.

The Mg–CO_2_ battery with ethanol (200 ppm) vapor exhibits the smallest discharge–charge voltage gap of 0.52 V at a cut-off discharge/charge capacity of 500 mAh g^−1^ and at a current density of 200 mA g^−1^ (Fig. 2a and Table S2), indicating a rapid decrease in resistance and electrode polarization. The reduced voltage gap effectively mitigates parasitic reactions induced by high overpotentials, thereby enhancing charge/discharge reversibility and improving cycling performance. Additionally, the Mg–CO_2_ battery with ethanol (200 ppm) vapor showcases the most stable operation over 600 h, without any capacity degradation (Fig. 2b). The tight voltage gap and extended operating lifespan highlight the excellent electrochemical performance of the suitable ethanol vapor in facilitating the reversible deposition and decomposition of discharge products in the Mg–CO_2_ battery. The Mg–CO_2_ battery with ethanol (200 ppm) vapor achieves a remarkably high full discharge capacity of 50 000 mAh g^−1^, further demonstrating its excellent electrochemical reversibility (Fig. 2c).

Moreover, the Mg–CO_2_ battery with 200 ppm ethanol vapor demonstrates excellent rate capability, as evidenced by the flat and well-defined charge/discharge plateaus across a wide current-density range (Fig. 2d and Table S3). Even under a high current density of 1600 mA g^−1^, the voltage gap remains within 1.2 V, indicating low polarization and robust kinetics (Fig. 2d and Figs S1 and S2). The superior electrochemical reversibility is further supported by cyclic voltammetry (CV) at 1 mV s^−1^, in which the battery with ethanol vapor exhibits the highest discharge onset potential (∼0.9 V for CO_2_RR) and the lowest charge onset potential (∼1.4 V for CO_2_RR), confirming its enhanced ability to drive both CO_2_ reduction and product decomposition efficiently (Fig. 2e).

The charge-transfer resistance (*R*_ct_) of the Mg–CO_2_ batteries with varying ethanol vapor contents was tested through electrochemical impedance spectroscopy (EIS) (Fig. S3 and Table S4) [20–22]. The Mg–CO_2_ battery with ethanol (200 ppm) vapor exhibits the lowest *R*_ct_ of 1589 Ω after discharge, reflecting the formation of discharge products with favorable electronic and ionic conductivity. Upon recharging, its *R*_ct_ nearly returns to the pristine level, indicating the complete decomposition of discharge products and excellent cycling reversibility. In stark contrast, the *R*_ct_ of the Mg–CO_2_ battery without ethanol remains nearly nine times higher than its initial value after recharging, revealing persistent accumulation of insulating products and poor reversibility (Table S5).

To verify that the discharge capacity originates solely from CO_2_ reduction, a control battery (Mg–Ar) was assembled with 200 ppm of ethanol vapor in an Ar atmosphere. This configuration delivered negligible discharge/charge capacity (Figs S4 and S5), indicating that the electrolyte remains stable within the operating voltage range and the capacity in Mg–CO_2_ batteries stems exclusively from CO_2_RR/CO_2_ER. Further support comes from linear-sweep voltammetry, which shows no notable current response until 4.65 V, confirming that electrolyte decomposition only initiates beyond this voltage and does not interfere with normal operation (Fig. S6). To decouple the CO_2_RR and CO_2_ER steps, a Mg–CO_2_ battery was first discharged in a CO_2_ atmosphere, then recharged in Ar with 200 ppm of ethanol vapor. The battery displayed similar charging profiles and overpotentials to those recharged in CO_2_, suggesting that CO_2_ is not required for the CO_2_ER process. However, the subsequent discharge curve in Ar showed a lower voltage plateau due to limited CO_2_ availability (Figs S7 and S8), confirming that CO_2_ is essential for sustained reduction reactions.

### Characterization of products in Mg–CO_2_ batteries

**Figure 3.**
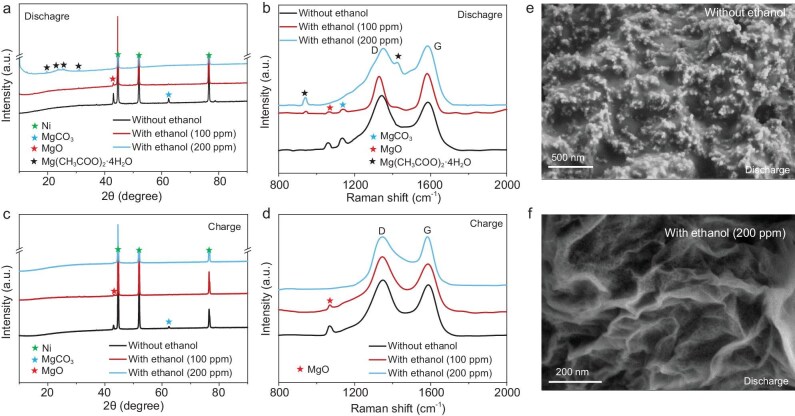
Discharge product analysis of Mg–CO_2_ batteries. (a) XRD patterns after discharge, (b) Raman spectroscopy after discharge, (c) XRD patterns after charge, (d) Raman spectroscopy after charge of the graphene cathode with different contents of ethanol. SEM images of the graphene cathodes (e) without ethanol and (f) with ethanol (200 ppm) vapor.

X-ray diffraction (XRD) analysis demonstrates a clear ethanol-induced regulation of CO_2_RR pathways. Without ethanol vapor, the discharge products of the Mg–CO_2_ battery are identified as MgO (JCPDS No. 45–0946) and MgCO_3_ (JCPDS No. 08–0479), corresponding to the conventional reaction route (Fig. 3a). Upon introducing ethanol (100 ppm) vapor, a new product, Mg(CH_3_COO)_2_·4H_2_O (JCPDS No. 00–011–0709), begins to appear alongside MgO and MgCO_3_, indicating a shift in the reaction pathway. Notably, when the ethanol concentration is increased to 200 ppm, Mg(CH_3_COO)_2_·4H_2_O becomes the sole crystalline product, confirming the dominant role of ethanol in steering the discharge reaction toward an alternative acetate-based pathway. This product transformation is further corroborated by using Raman spectroscopy (Fig. 3b), which reveals the characteristic vibrational peaks of Mg(CH_3_COO)_2_·4H_2_O. This identification can be further substantiated by using Fourier transform infrared (FTIR) and X-ray photoelectron spectroscopy (XPS) analyses (Figs S9 and S10), which provide additional chemical evidence. These findings highlight the ability of ethanol vapor to effectively reconfigure the CO_2_RR pathway by selectively stabilizing acetate intermediates, enabling precise control over discharge product composition.

XRD analysis reveals that, after charging, the characteristic peaks of Mg(CH_3_COO)_2_·4H_2_O completely disappear from the graphene cathode discharged in ethanol (200 ppm) vapor, indicating its full decomposition (Fig. 3c). Raman spectra further confirm this complete removal of Mg(CH_3_COO)_2_·4H_2_O under the same charging conditions (Fig. 3d). In contrast, for the Mg–CO_2_ batteries discharged without ethanol or with low ethanol content (<200 ppm), residual peaks of MgO and MgCO_3_ remain after charging, suggesting incomplete decomposition. These results demonstrate the exceptional electrochemical reversibility of Mg(CH_3_COO)_2_·4H_2_O relative to the refractory MgO and MgCO_3_.

The cathode retrieved from a battery discharged to a specific capacity of 500 mAh g^−1^ in a CO_2_ atmosphere without ethanol vapor exhibits a thick and dense layer of MgO and MgCO_3_ as the predominant discharge products (Fig. 3e). This compact deposit severely impedes CO_2_ electroreduction reversibility, increases charge-transfer resistance and intensifies cathode polarization. With each cycle, the persistent accumulation of this insulating layer ultimately leads to battery failure. In contrast, the formation of such dense deposits is significantly mitigated with increasing ethanol vapor concentrations (Fig. S11). At an ethanol vapor concentration of 200 ppm, the discharge products primarily adopt a thin-film or small-particle morphology, identified as Mg(CH_3_COO)_2_·4H_2_O (Fig. 3f). This morphology facilitates more uniform access of both CO_2_ and electrolyte to active material, enhances charge-transfer kinetics and promotes the efficient decomposition of Mg(CH_3_COO)_2_·4H_2_O during the CO_2_ER process, thereby enabling superior cycling reversibility.

**Figure 4.**
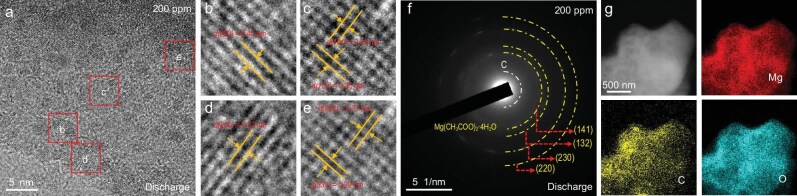
Discharge product analysis of Mg–CO_2_ batteries. (a) HR-TEM images of the graphene cathode with ethanol (200 ppm) vapor after the discharging. (b–e) Interfacial Fourier transform technique (IFTT) patterns of the corresponding regions (b), (c), (d) and (e), respectively, marked in (a). (f) SAED pattern of the graphene cathode with ethanol (200 ppm) vapor after the discharging. (g) Elemental mappings of Mg, C and O of the graphene cathode with ethanol (200 ppm) vapor after the discharging.

High-resolution transmission electron microscopy (HR-TEM) images (Fig. 4a) vividly capture the evolution of the cathode surface during electrochemical discharge in a CO_2_ atmosphere containing 200 ppm of ethanol vapor. At the end of discharging, a well-defined crystalline layer of Mg(CH_3_COO)_2_·4H_2_O was formed on the graphene surface, indicating the ethanol-assisted product formation. Furthermore, the fast Fourier transform patterns and inverse fast Fourier transform images obtained from selected regions within the crystalline layer (Fig. 4b–e) reveal that the distance between adjacent lattice planes of the particles corresponds to that of Mg(CH_3_COO)_2_·4H_2_O. Additionally, the diffraction pattern of discharge product in the selected area electron diffraction (SAED) further confirms this conclusion (Fig. 4f). The uniform distribution of Mg(CH_3_COO)_2_·4H_2_O on the graphene is demonstrated through scanning transmission electron microscopy (STEM) images and energy-dispersive X-ray spectroscopy (EDS) mappings of Mg, O and C (Fig. 4g and Fig. S12), indicating the formation of uniform thin-film/small-particle crystals on the surface of the cathode.

Scanning electron microscopy (SEM), TEM, and SAED analyses confirm that the thin-film/small-particle Mg(CH_3_COO)_2_·4H_2_O layer completely disappears after charging in the presence of 200 ppm of ethanol vapor (Fig. S13), underscoring the excellent reversibility enabled by its favorable morphology. In stark contrast, the dense layer of MgO and MgCO_3_ formed in the absence of ethanol persists on the graphene cathode surface even after charging (Fig. S14). These residual products form an insulating barrier that obstructs ion and electron transport at the electrode–electrolyte interface, prematurely terminating the charge process and significantly compromising reversibility. The introduction of ethanol vapor effectively prevents the formation of such passivating deposits, thereby ensuring sustained electrochemical activity and stable cycling performance.

To investigate the long-term effect of ethanol vapor on the discharge product, the SEM and XRD results of the Mg–CO_2_ battery with 200 ppm of ethanol vapor after the 50th cycle were studied. The SEM results show the formation of the thin-film/small-particle Mg(CH_3_COO)_2_·4H_2_O structure over the battery with ethanol vapor (200 ppm) after the discharge process (Fig. S15a) and disappear after the charge process at the 50th cycle (Fig. S15b). Additionally, XRD results confirm the reversible formation and decomposition of Mg(CH_3_COO)_2_·4H_2_O during discharge and charge at the 50th cycle (Fig. S16). These results indicate that ethanol vapor persistently modulates the surface reaction pathway toward the formation of thin-film/small-particle Mg(CH_3_COO)_2_·4H_2_O, thereby ensuring electrochemical reversibility.

### Electrochemical reaction mechanism

**Figure 5.**
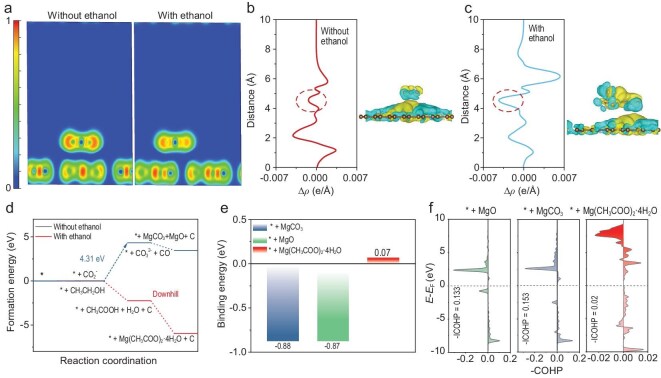
Reaction mechanism analysis of Mg–CO_2_ batteries. (a) ELFs of *CO_2_–graphene without ethanol and with ethanol. Charge density difference of *CO_2_–graphene (b) without and (c) with ethanol. Cyan and yellow regions denote electron depletion and accumulation, respectively. (d) Reaction pathway with/without ethanol. (e) Binding energies of different discharge products. (f) COHP of MgO, MgCO_3_ and Mg(CH_3_COO)_2_·4H_2_O with substrate, respectively.

To investigate the effect of ethanol vapor on the discharge mechanism of Mg–CO_2_ batteries, density functional theory (DFT) calculations were performed. Upon ethanol introduction, the CO_2_ adsorption energy on the graphene increases from −0.16 to −0.39 eV (Fig. S17 and Table S6), indicating a stronger CO_2_–substrate interaction. Electron localization function (ELF) analysis shows that the presence of ethanol reduces electron confinement near the CO_2_ molecule, thereby facilitating charge transfer to the substrate (Fig. 5a). Compared with the interface without ethanol (0.001 e/Å), the presence of ethanol markedly enhances electron transfer at the interface, as evidenced by an increased electron-transfer value of 0.004 e/Å (Fig. 5b and c). This interfacial modulation significantly alters the reaction pathway. Without ethanol, CO_2_ reduction proceeds toward MgO and MgCO_3_ formation, requiring a high energy barrier of 4.31 eV (Fig. 5d). In contrast, ethanol vapor shifts the product toward Mg(CH_3_COO)_2_·4H_2_O, with all intermediate steps exhibiting exothermic energy profiles, which not only avoids the formation of insulating byproducts, but also contributes to enhanced reversibility.

The detachment of discharge products is critical for battery reversibility. Among the possible species, Mg(CH_3_COO)_2_·4H_2_O exhibits the weakest binding affinity to the graphene substrate, with a calculated adsorption energy of only 0.07 eV, in sharp contrast to MgO (−0.87 eV) and MgCO_3_ (−0.88 eV) (Fig. 5e and Figs S18–S20). This weak interfacial interaction facilitates easier detachment and decomposition of Mg(CH_3_COO)_2_·4H_2_O during the charging process. This trend is further supported by using crystal orbital Hamilton population (COHP) analysis, where Mg(CH_3_COO)_2_·4H_2_O shows a significantly weaker bonding strength with the substrate (−0.017 eV) compared with MgO (−0.133 eV) and MgCO_3_ (−0.153 eV) (Fig. 5f). Bader charge analysis and differential charge density (Figs S21 and S22) further reveal that Mg(CH_3_COO)_2_·4H_2_O induces the lowest degree of electron accumulation (−0.17 |e|) on the graphene surface, indicating minimal electronic coupling with the substrate. Such weak binding not only promotes the facile removal of discharge products upon charging, but also mitigates the risk of interfacial passivation. Additionally, density-of-states calculation indicates that Mg(CH_3_COO)_2_·4H_2_O has a narrower bandgap relative to MgO and MgCO_3_ (Fig. S23), implying higher electronic conductivity. The enhanced conductivity could promote electron transfer during charge/discharge, improving the reaction kinetics and overall battery performance.

## CONCLUSIONS

In summary, we pioneer an ethanol vapor-mediated strategy that fundamentally reshapes the reaction pathway of Mg–CO_2_ batteries, shifting the discharge product from inert MgO and MgCO_3_ to an unreported highly conductive C_2+_ species, Mg(CH_3_COO)_2_·4H_2_O. This paradigm shift transforms the reaction landscape, circumventing the high energy barrier of 4.31 eV associated with conventional products and instead proceeding through an exothermic downhill process. DFT calculations unveil that Mg(CH_3_COO)_2_·4H_2_O exhibits an exceptionally weak interaction with the graphene substrate (−0.017 eV), drastically lowering the kinetic barriers of CO_2_RR/CO_2_ER and enabling fast reaction dynamics. As a result, the engineered Mg–CO_2_ battery achieves record-breaking performance, with both discharge and charge capacities surpassing 50 000 mAh g^−1^ and ultra-stable operation over 600 h. This work not only redefines the reaction paradigm of Mg–CO_2_ systems, but also offers a generalizable gas-phase reactant design principle to unlock next-generation carbon fixation and energy-conversion technologies.

## METHODS

### Preparation of Mg–CO_2_ batteries

The graphene catalyst, glass fiber separator (GF/A, Whatman), electrolyte (0.05 M MgCl_2_, 0.5 M Mg(TFSI)_2_ in tetraethylene glycol dimethyl ether (TEGDME)) and Mg metal foil (thickness, 0.5 mm) were assembled in a CR2032 coin-type cell. The loading of the graphene cathode was ∼0.2 mg. In this work, all current densities and specific capacities were normalized to the mass of the graphene cathode. All the cells were assembled in an Ar-filled glovebox (the contents of water and oxygen were <0.1 ppm). Electrochemical tests were carried out in homemade bottles filled with CO_2_. Different contents of alcohol (0, 50, 100, 200, 400 and 800 ppm) were dropped into the bottles for the evaporation. To avoid the exhaustion of the solvent, a vial with 200 μL of TEGDME was put in as well. Before testing, the cells were placed in homemade bottles filled with CO_2_ to allow stabilization for 8 h.

### Electrochemical measurements

At room temperature, the charge/discharge termination condition was either through a cut-off specific capacity of 1000 mAh g^−1^ or a cut-off discharge/charge voltage of 0.1 V/4.8 V, respectively. The discharge–charge curves at different current densities were obtained by using a NEWARE Cell Tester. The EIS was conducted on an Ivium electrochemical workstation from 10^6^ to 0.1 Hz. The CV was tested on the Ivium electrochemical workstation from 0.1 to 4.8 V.

### Physical characterization

SEM images and SEM–EDS were taken on a JEOL JSM-6701F Field-Emission microscope. HR-TEM images were taken on a JEM-2200FS JEOL. Raman spectra were obtained on a Micro-Raman spectrophotometer (MonoRa750i, Optron). The carbide materials and air electrodes after the cycling test were measured by using the X-ray diffraction instrument (XRD, D/MAX 2500, Rigaku) with a Cu Kα radiation source within 2θ of 10.0° to 90.0° at a scan rate of 5° min^−1^. The FTIR spectra were obtained by using a Nicolet iS10 FTIR Spectrometer (Thermo Scientific) in a range of 400–4000 cm^−1^. The cycled Mg electrodes were characterized by using SEM, focused-ion-beam-assisted scanning electron microscopy (FIB-SEM) and XPS. The XPS measurement was taken on an ESCALAB 250Xi, Thermo Scientific. The batteries for the *ex situ* characterizations were disassembled in a glovebox after discharge/charge.

### Computational method

We employed the Vienna Ab initio Simulation Package (VASP, Version 5.44) to perform all DFT calculations with the generalized gradient approximation by using the Perdew–Burke–Ernzerhof functional [23–25]. We have chosen the projected augmented wave potentials to describe the ionic cores and take valence electrons into account by using a plane wave basis set with a kinetic energy cut-off of 450 eV [26]. Geometry optimizations were performed with a force convergence of <0.05 eV/Å. To ensure sufficient spacing, we placed a vacuum spacing of 15 Å perpendicular to the surface. The original bulk structures of Mg(CH_3_COO)_2_·4H_2_O were optimized before the construction of surfaces with a Gamma Scheme k-point of 3 × 2 × 2. Gamma Scheme k-points of 2 × 2 × 1, 6 × 4 × 1 and 1 × 2 × 1 were applied for the MgCO_3_ (122) surface, MgO (220) surface and Mg(CH_3_COO)_2_·4H_2_O (220) surface, respectively. After geometry optimization, the plots of projected density of states were calculated by using the same parameter method.

## Supplementary Material

nwaf330_Supplemental_File
