# Supplementary material for: Ethanol vapor-driven multicarbon chemistry enables high-performance Mg–CO2 battery
Source: Natl Sci Rev. 2025 Aug 12;12(10):nwaf330. doi: 10.1093/nsr/nwaf330 (PMC12492234; doi:10.1093/nsr/nwaf330)
Supplement: nwaf330_Supplemental_File [file nwaf330_supplemental_file.pdf]

Supporting Information

Ethanol Vapor-Driven Multi-Carbon Chemistry Enables High-Performance Mg-CO_2 Battery

Wenbo Liu[1, 2+], Lu Li[2+], Menggang Li[2+], Ning Wang[1], Yanmei Li[1], Zongqiang Sun[2], Youxing Liu[2], Mingyang Chen[1], Rui Xu[1]*, Shaojun Guo[2]*

[1]School of Materials Science and Engineering, University of Science and Technology Beijing, Beijing 100083, China.

[2]School of Materials Science and Engineering, Peking University, Beijing 100871, China.

[+]Equally contributed to this work.

Correspondence should be addressed to Rui Xu; ruixu@ustb.edu.cn, and Shaojun Guo; guosj@pku.edu.cn.

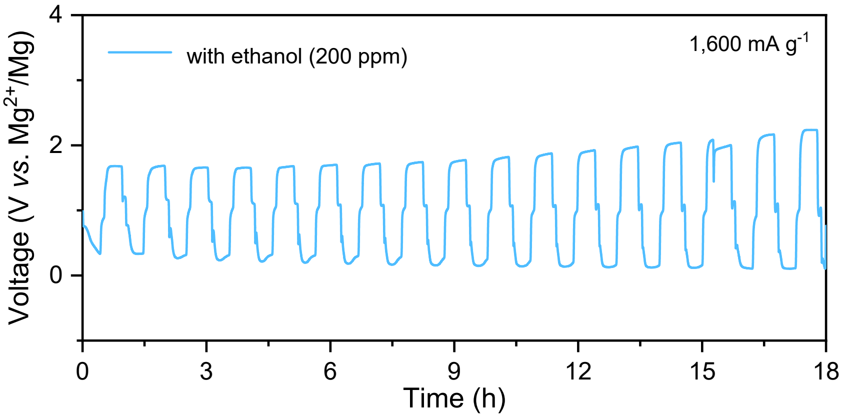

Figure S1. The voltage-time curves of $Mg\text{-}CO_2$ battery with ethanol (200 ppm) vapor under the current density of 1,600 mA g^{-1} with a cut-off specific capacity of 500 mAh g^{-1}.

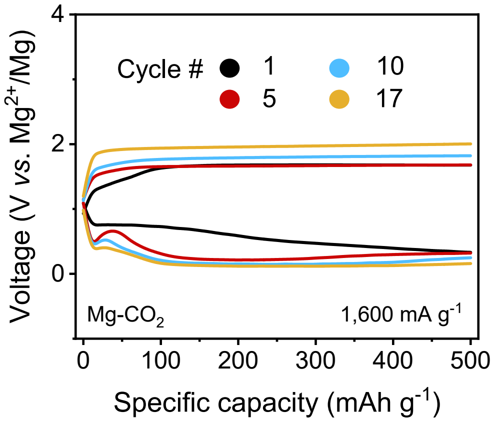

Figure S2. The discharge-charge profiles of Mg-CO_2 battery with ethanol (200 ppm) vapor under the current density of 1,600 mA g^{-1} with a cut-off specific capacity of 500 mAh g^{-1}.

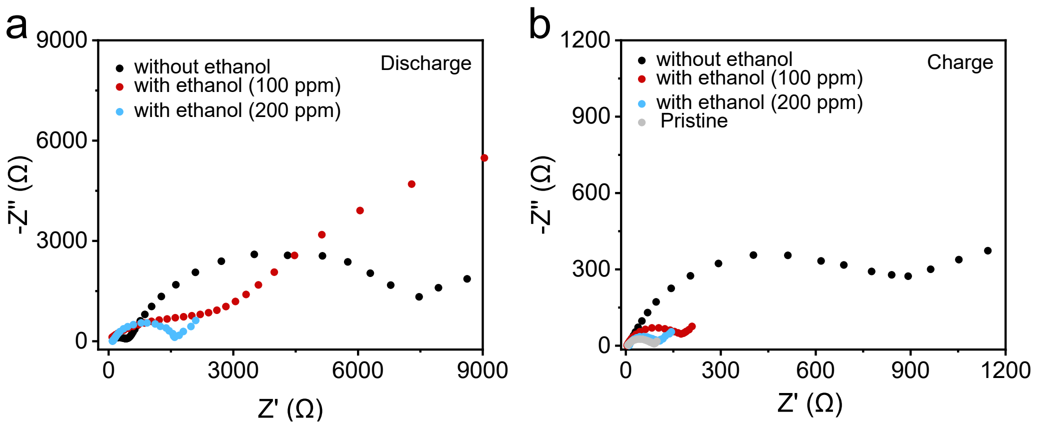

Figure S3. (a) EIS results of Mg-CO_2 batteries by adding different-content ethanol after the discharge. (b) EIS results of Mg-CO_2 batteries by adding different-content ethanol after the charge and the pristine states, respectively.

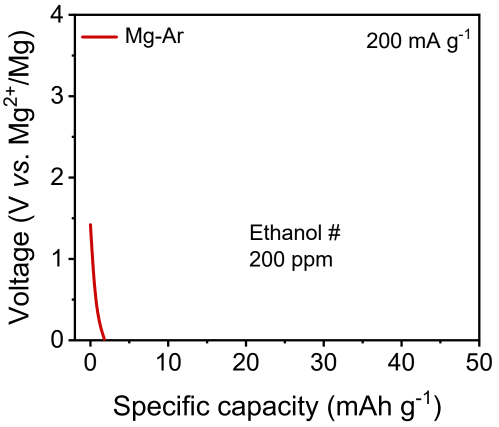

Figure S4. The discharge profiles of Mg-Ar battery with the addition of 200 ppm ethanol into Ar atmosphere under the current density of 200 mA g^{-1} with a cut-off specific capacity of 500 mAh g^{-1} and under a cut-off discharge voltage of 0.1 V.

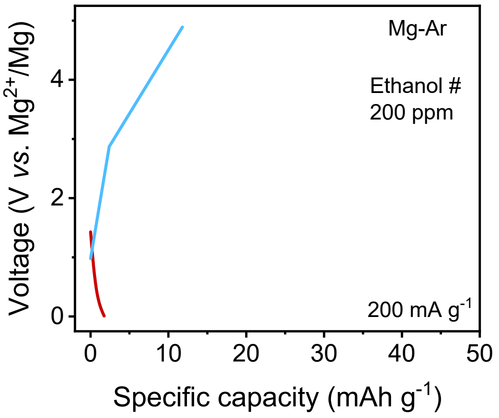

Figure S5. The discharge-charge profiles of Mg-Ar battery with the addition of 200 ppm ethanol into Ar atmosphere under the current density of 200 mA g^{-1} with a cut-off specific capacity of 500 mAh g^{-1} and under a cut-off discharge and charge voltage of 0.1 V and 4.8 V, respectively.

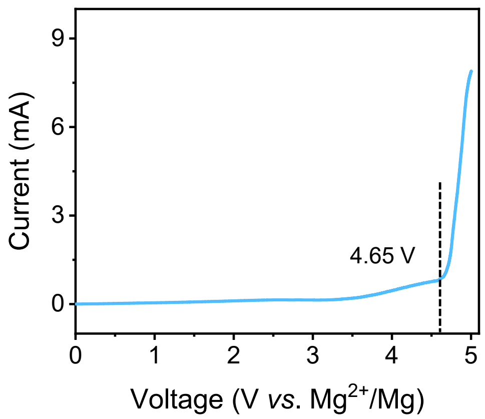

Figure S6. Oxidation behavior of electrolyte (0.5 M $Mg(TFSI)_2$, 0.05 M $MgCl_2$ in TEGDME) by linear sweep voltammetry (LSV) with stainless steel gaskets as both the working and counter electrode.

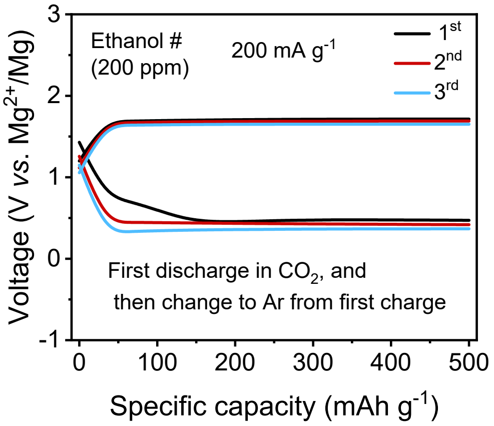

Figure S7. The discharge-charge voltage profiles of the battery initially discharged in carbon dioxide atmosphere with added ethanol (200 ppm) and then transferred to argon atmosphere with the addition of ethanol (200 ppm) for subsequent charge-discharge test under the current density of 200 mA g^{-1} with a cut-off specific capacity of 500 mAh g^{-1}.

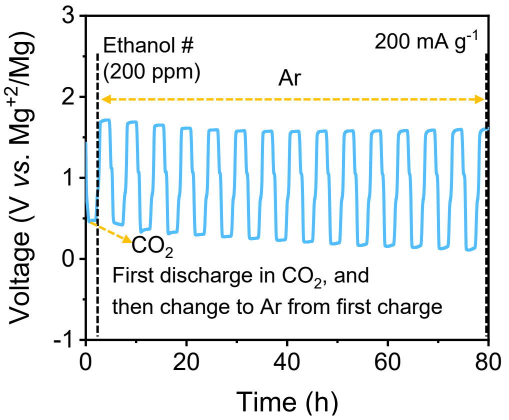

Figure S8. The voltage-time curves of the battery discharged in carbon dioxide atmosphere with the addition of ethanol (200 ppm), and then transferred to argon atmosphere with the addition of ethanol (200 ppm) for subsequent charge-discharge test under the current density of 200 mA g^{-1} with a cut-off specific capacity of 500 mAh g^{-1}.

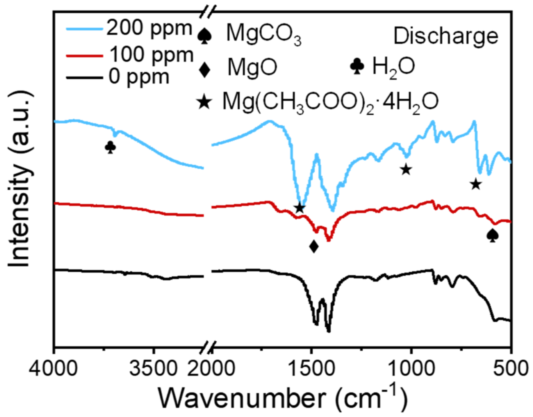

Figure S9. FTIR spectra of the graphene electrode with different-content ethanol (0 ppm, 100 ppm and 200 ppm).

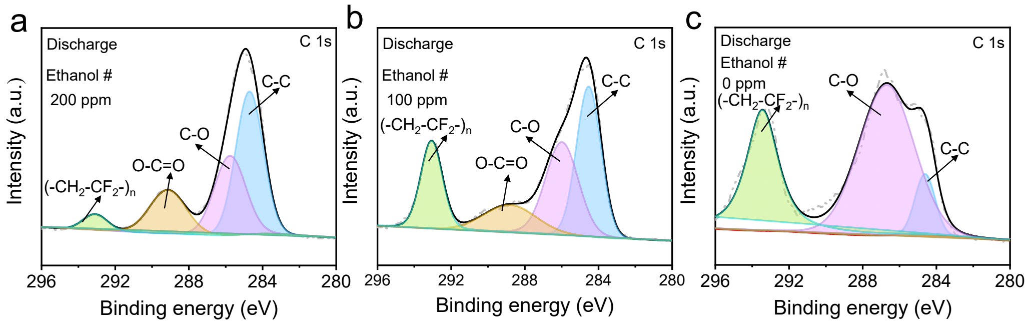

Figure S10. XPS of C 1s for graphene electrode with different-content ethanol (0 ppm, 50 ppm, 100 ppm and 200 ppm ethanol) after the discharge. (a) 200 ppm, (b) 100 ppm and (c) 0 ppm.

To gain further insight into the components of discharge products, X-ray photoelectron spectroscopy (XPS) was carried out to analyze the oxidation state and composition of the surface for graphene electrode, which was tested in CO_2 atmosphere with the addition of 0 ppm, 50 ppm, 100 ppm and 200 ppm ethanol, respectively. Figure S10a shows the C 1s spectra for the graphene cathode tested in CO_2 atmosphere containing 200 ppm ethanol. A new peak was located at around 289.0 eV (corresponding to O-C=O) after the discharge, attributed to $Mg(CH_3COO)_2{\cdot}4H_2O$[1, 2]. The peak located at ~293.0 eV (corresponding to $(-CH_2-CF_2-)n$) was originated from the electrolyte, and the peak located at ~284.7 eV (corresponding to C-C) stemmed from the graphene electrode itself [3]. While for the graphene cathode tested in CO_2 atmosphere containing 0 ppm or 100 ppm ethanol, all of which showed a high peak located at around 286.8 eV (C-O), attributable to $MgCO_3$[4, 5], and a low peak located at around 289.0 eV (O-C=O), as shown in Fig. S10.

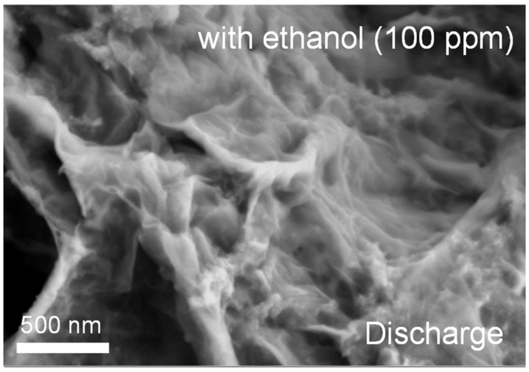

Figure S11. SEM images of the graphene cathode with ethanol (100 ppm) vapor.

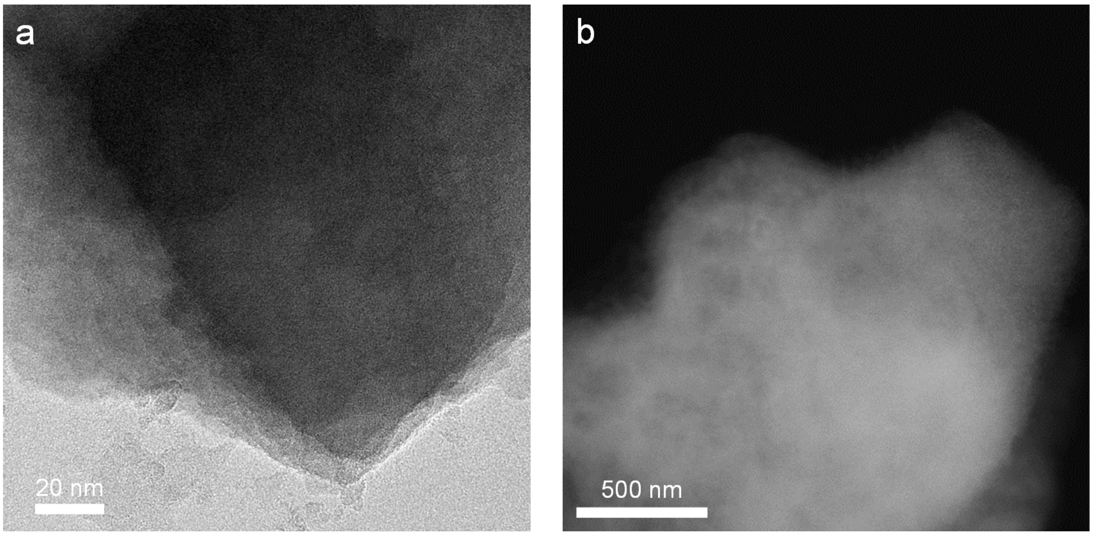

Figure S12. (a) TEM and (b) STEM images of the Mg-CO_2 battery with ethanol (200 ppm) vapor at the discharge.

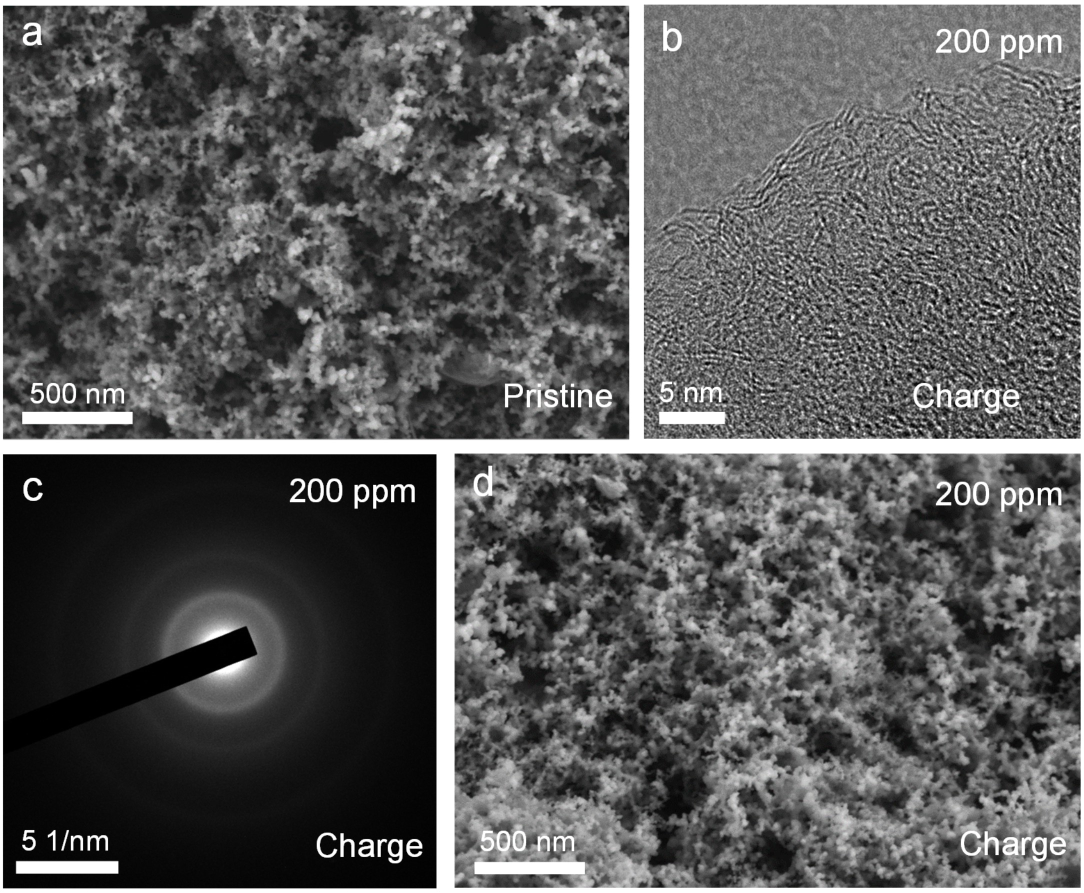

Figure S13. (a) SEM image of the pristine graphene electrode. (b) HR-TEM images of graphene electrode with the addition of 200 ppm ethanol after the charge. (c) SAED pattern of graphene electrode with the addition of 200 ppm ethanol after the charge. (d) SEM image of the charge graphene electrode.

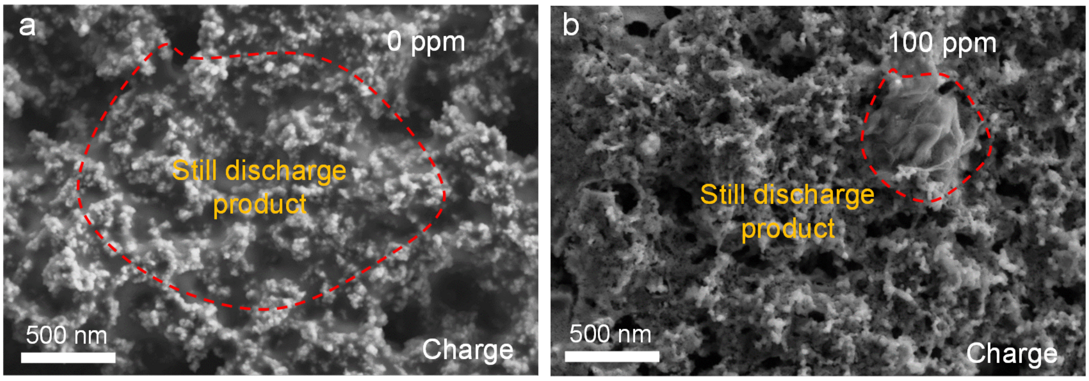

Figure S14. SEM image of the graphene cathodes of (a) without ethanol, (b) with ethanol (100 ppm) vapor after the charge.

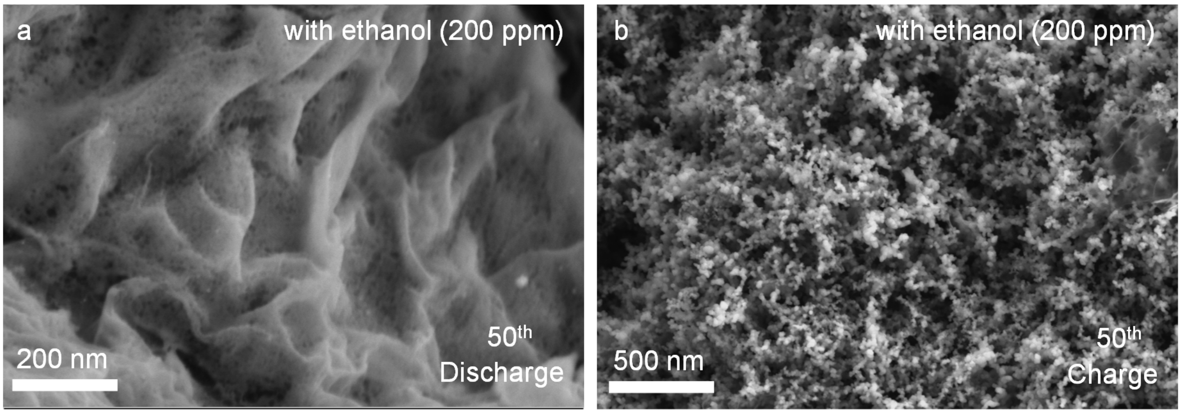

Figure S15. SEM images of cathode for Mg-CO_2 battery with ethanol (200 ppm) after 50th cycles. (a) after discharge stage and (b) after charge stage.

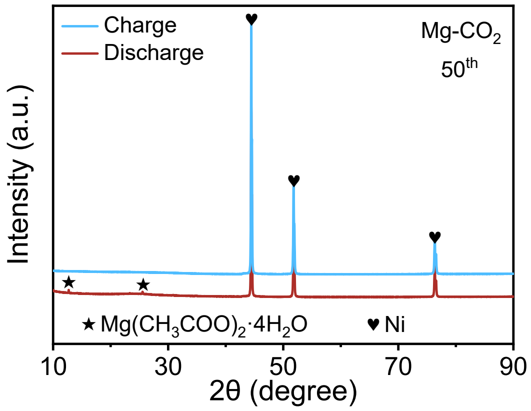

Figure S16. XRD patterns of Mg-CO_2 battery with ethanol (200 ppm) at discharge and charge after 50th cycles.

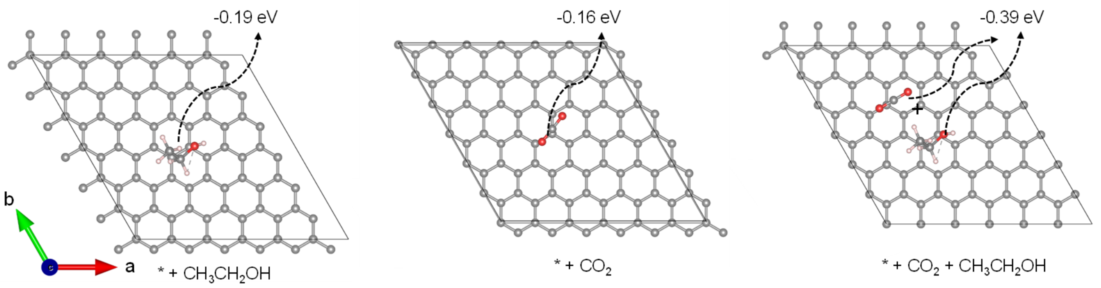

Figure S17. The adsorption energy of * + CO_2, * + CH_3CH_2OH, and * + CO_2 + CH_3CH_2OH on graphene substrate, respectively.

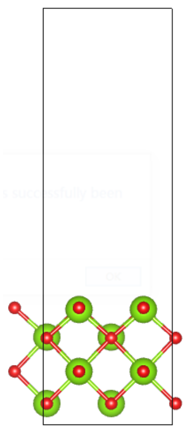

Figure S18. The optimized structure of MgO.

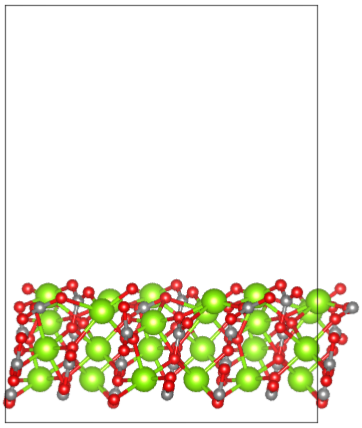

Figure S19. The optimized structure of $MgCO_3$.

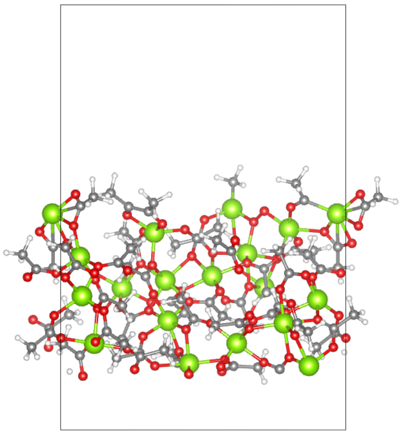

Figure S20. The optimized structure of $Mg(CH_3COO)_2 \cdot 4H_2O$.

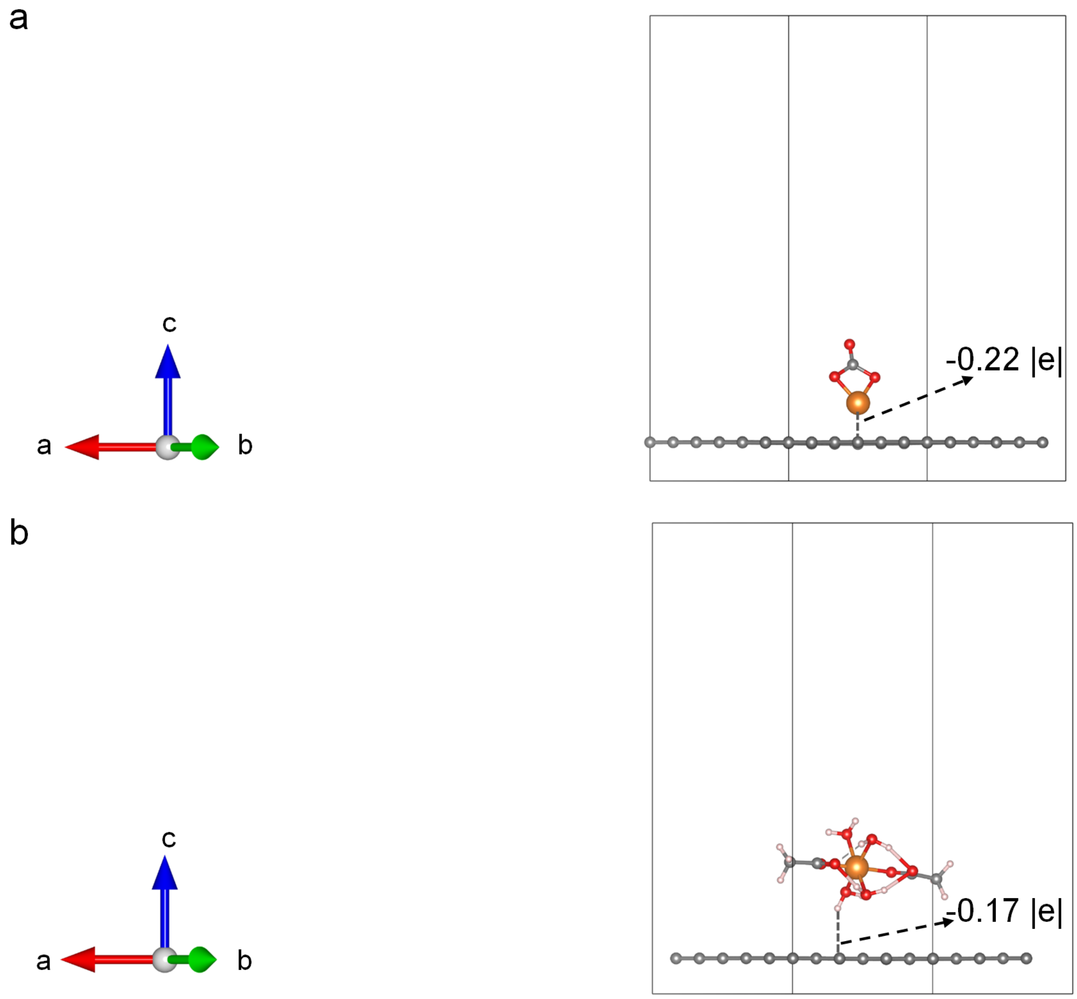

Figure S21. Bader charge results of (a) $MgCO_3$ and (b) $Mg(CH_3COO)_2{\cdot}4H_2O$ on the graphene surface, respectively.

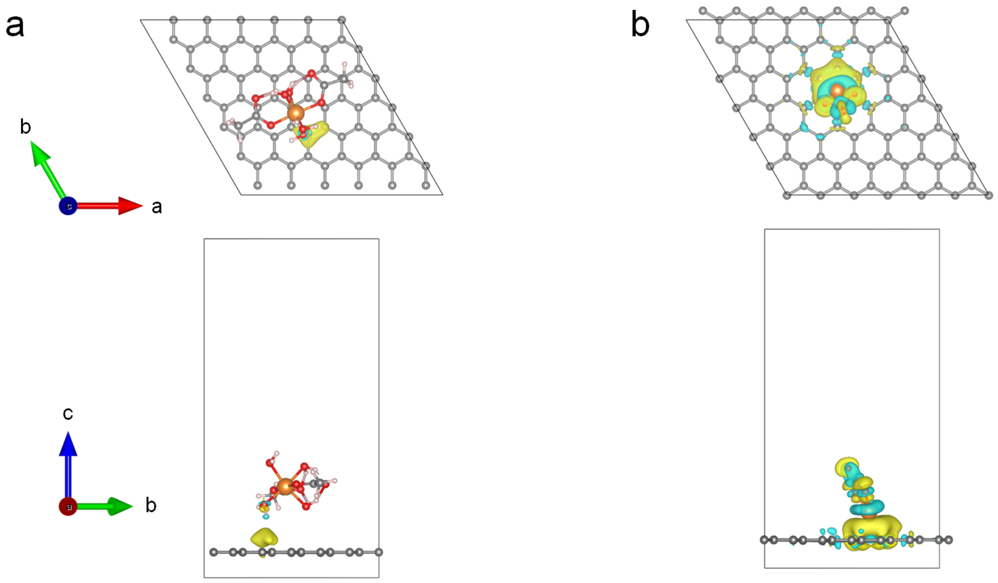

Figure S22. Differential charge density upon the adsorption of (a) $Mg(CH_3COO)_2{\cdot}4H_2O$ and (b) $MgCO_3$. Both displayed on the same iso-surface. Yellow and blue iso-surfaces represent charge accumulation and depletion, respectively.

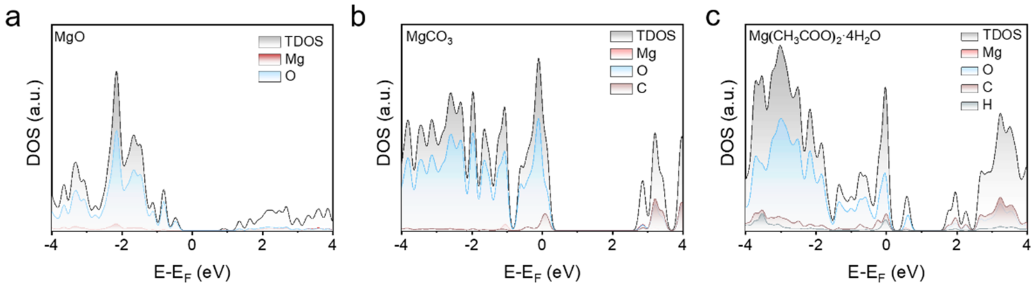

Figure S23. The projected DOS of (a) MgO. (b) $MgCO_3$ and (c) $Mg(CH_3COO)_2{\cdot}4H_2O$.

Table S1. The electrochemical performance comparisons of our developed Mg-CO_2 batteries with the previously reported ones.

Catalyst	Specific capacity (mAh g^{-1})	Overpotential (V)	Temperature (°C)	Current density (mA g^{-1})	Catalyst loading (mg)
PDA	1,000	1.5	Room temperature	200	0.1
CNTs	5,000	2.6	Room temperature	500	0.1 or 0.05
Mo_2C-CNTs	4,103.8	4.7	Room temperature	5	0.4
Mo_2C-NDs@CNF	4,398	4.7	Room temperature	200	0.2
$Mo_{4/3}(B_nN_{1-n})_{2-m}T_z$	10,300	2.6	Room temperature	100	0.2-0.25
Our work	50,000	0.66	Room temperature	400	0.2

Table S2. The overpotential and cycle life of Mg-CO_2 batteries tested in CO_2 atmosphere containing 0, 50, 100, 200, 400 and 800 ppm ethanol, respectively.

Cell	Discharge voltage (V)	Charge voltage (V)	Overpotential (V)	Cycle life (h)
0 ppm	0.3	3.6	3.3	< 5
50 ppm	0.3	3.0	2.7	< 35
100 ppm	0.6	2.8	2.3	< 41
200 ppm	0.88	1.4	0.52	> 600
400 ppm	0.6	2.6	2.0	< 42
800 ppm	0.4	2.6	2.2	< 21

Table S3. The overpotentials of Mg-CO_2 batteries tested in CO_2 atmosphere containing 200 ppm ethanol under different current densities.

Current density (mA g^{-1})	Discharge voltage (V)	Charge voltage (V)	Overpotential (V)	Cycle life (h)
200	0.88	1.4	0.52	> 250
400	0.6	1.6	1.0	> 250
800	0.6	1.7	1.1	
1,200	0.6	1.8	1.2	
1,600	0.5	1.7	1.2	

Table S4. Impedance parameters of Mg-CO_2 batteries with graphene cathode after the discharge tested in CO_2 atmosphere containing 0, 100 and 200 ppm ethanol, respectively.

Cell	0 ppm	100 ppm	200 ppm
R_s	464.0	87.4	87.4
R_f	7180.0	2963.0	1501.0
R_{ct}	7649.0	3052.0	1589.0

Table S5. Impedance parameters of Mg-CO_2 batteries with graphene cathode after the charge tested in CO_2 atmosphere containing 0, 100, 200 ppm ethanol, respectively.

Cell	0 ppm	100 ppm	200 ppm
R_s	7.4	6.6	6.5
R_f	885.0	166.8	91.2
R_{ct}	892.0	174.5	98.2

Table S6. The adsorption energies of CO_2, CH_3CH_2OH, CO_2 + CH_3CH_2OH on graphene substrate, respectively.

	E(total)	E(slab)	E(reference)	ΔE(eV)
* + $C_2H_6O_2$	-713.95	-666.83	-46.93	-0.19
* + CO_2	-689.95	-666.83	-22.96	-0.16
* + CO_2 + $C_2H_6O_2$	-737.11	-666.83	-69.89	-0.39

$\Delta E(* + C_2H_6O) = E(slab + C_2H_6O) - E(slab) - E(C_2H_6O)$

$\Delta E(* + CO_2) = E(slab + CO_2) - E(slab) - E(CO_2)$

$\Delta E(* + CO_2 + C_2H_6O) = E(slab + CO_2 + C_2H_6O) - E(slab) - E(C_2H_6O) - E(CO_2)$

References

1. Ryu JS, Lim ET, Choi JS *et al.* Dry etching of copper thin films in high density plasma of CH_3COOH/Ar. *Thin Solid Films*. 2019; **672**: 55-61.
2. Chen F, Chen G, Huang P *et al.* High surface area porous carbon derived from chitosan: $CH_3COOH/(NH_4)_2HPO_4$ dual-assisted hydrothermal carbonization synthesis and its application in supercapacitor. *J Energy Storage*. 2023; **71**: 108180.
3. Qi G, Zhang J, Chen L *et al.* Binder-free MoN nanofibers catalysts for flexible 2-electron oxalate-based Li-CO_2 batteries with high energy efficiency. *Adv Funct Mater*. 2022; **32**: 2112501.
4. Li J, Zhang K, Zhao Y *et al.* High-Efficiency and Stable Li-CO_2 Battery Enabled by Carbon Nanotube/Carbon Nitride Heterostructured Photocathode. *Angew Chem Int Ed*. 2022; **61**: e202114612.
5. Lu Y, Zhao C, Khanal S *et al.* Controllable synthesis of hierarchical nanostructured anhydrous $MgCO_3$ and its effect on mechanical and thermal properties of PVC composites. *Compos Part A-Appl S*. 2020; **135**: 105926.
